# Cutaneous B-cell Pseudolymphoma: A Rare Case Masquerading a Thoracic Mass in a Fourteen-Year-Old Male Patient

**DOI:** 10.7759/cureus.38003

**Published:** 2023-04-23

**Authors:** Chrysovalanti Oikonomidi, Maria Troupi, Leonidas Marinos, Dimitris Liatsos, Dimosthenis Chrysikos, Dimitrios Filippou, Theodore Troupis

**Affiliations:** 1 Department of Anatomy, National and Kapodistrian University of Athens, Athens, GRC; 2 Department of Hematopathology, Evangelismos General Hospital, National and Kapodistrian University of Athens, Athens, GRC

**Keywords:** surgical treatment, immunohistochemistry, idiopathic, b-cell, cutaneous pseudolymphoma

## Abstract

Cutaneous B-cell pseudolymphoma (CBPL) may appear in the face, chest, or upper limbs, and it can be asymptomatic or in the form of nodules, papules, or masses. In most cases, it is idiopathic. However, some causes that have been identified are trauma, contact dermatitis, injected vaccinations, bacterial infections, tattoo dyes, insect bites, and certain drugs. Since the histology and clinical presentation of cutaneous pseudolymphoma (CPSL) are similar to those of cutaneous lymphomas, the diagnosis is usually based on an incisional or excisional biopsy. In this paper, a 14-year-old male patient with a two-month-old mass in the right lateral thoracic region is taken as a case study. He had neither symptoms, nor a past medical history, nor a family history. He had an insect bite a month ago and was fully vaccinated. However, the mass was some centimeters away from the insect bite. A biopsy was taken. The products of it were two paraffin cubes and two histological slides (H&E). The diagnosis was cutaneous B-cell pseudolymphoma. The total removal of the mass was decided since, in idiopathic cases like this, CBPL is not usually healed with topical and non-invasive treatments. Follow-up examinations were suggested since a further antigenic reaction is possible. If cutaneous B-pseudolymphoma is early diagnosed and treated, it does not cause serious problems. In some cases, it even resolves on its own.

## Introduction

Cutaneous B-cell pseudolymphoma (CBPL) encloses several terms, including lymphocytoma cutis, cutaneous lymphoid hyperplasia, sarcomatosis cutis of Spiegler-Fendt, and Bafverstedt syndrome [1,2]. CBPL is described in the literature as a lymphoproliferative reactive process in the skin, usually located in the face, chest, or upper limbs [2]. It is more commonly found in men, with a male-to-female ratio of 3:1, and in white individuals, with a white-to-black ratio of 9:1. The disease onset happens in early adulthood, with a median age of 34, and two-thirds of the patients are found to be under 40 years old [3,4]. The clinical presentation of CBPL shows wide differentiation, with the majority of cases being characterized by lesions similar to cutaneous B-cell lymphoma, that is, flesh-colored to plum-red cutaneous or subcutaneous nodules and plaques [4,5]. Lesions primarily affect the face, especially the nose, cheeks, and earlobe, followed by the upper trunk and the extremities [4]. Two forms have been described in the literature: the predominant localized form as a solitary or cluster of aggregated lesions, and the less frequent generalized form as multiple papules. The localized one appears in a variable color, from skin-colored to red-purple, and consists of asymptomatic nodules or tumors of up to 4 cm in diameter. Miliary papules can also be observed in specific clinical cases. Occasionally, constitutional symptoms may appear. Borrelia burgdorferi-associated cutaneous pseudolymphoma (CPSL), for example, sometimes presents with regional lymphadenopathy and is more rarely accompanied by Lyme disease constitutional symptoms [2,4,5].

In an attempt to define this heterogeneous pattern of the disease, various classification approaches have been suggested, taking into account its clinical and histologic presentation. More specifically, Mitteldorf and Kempf introduced the following four categories of the classification system for CPSLs: (1) Nodular PSLs, consisting of solitary or multiple nodules and bearing clinical and histologic resemblance to cutaneous lymphomas; (2) Mycosis fungoides and other cutaneous T-cell lymphomas (CTCLs) simulating PSLs that imitate mainly the histopathology of Mycosis fungoides or other CTCLs and include a large number of clinical entities; (3) other PSLs, a category containing discrete cases reported as PSL; (4) intravascular PSLs regarding reactive lymphocytes of atypical morphology accumulating inside small lymphatic vessels [6].

Even though the majority of the cases are considered idiopathic, several non-malignant stimuli have been attributed to causing CBPL. These stimuli can include trauma, contact dermatitis, injected vaccinations, bacterial infections, especially by Borrelia burgdorferi, viral infections by herpes simplex/zoster virus and HIV, tattoo dyes, and insect bites, but also certain drugs, including anticonvulsants, antibiotics, antipsychotics, anxiolytics, antirheumatics, antihypertensives, antiarrhythmics, and non-steroidal anti-inflammatory drugs. It is worth mentioning that idiopathic cases seem to be connected with photosensitivity, and tattoo-induced CBPLs primarily concern the usage of red dyes [1,2,4-7].

Clinical doctors encounter difficulties in diagnosing pseudolymphomas over lymphomas since the histology and clinical presentation of CPSL emulate cutaneous lymphomas. However, there are some epidemiological and clinical characteristics that can differentiate the clinical entities of primary cutaneous marginal zone lymphoma (PCMZL) and primary cutaneous follicle center lymphoma (PCFCL) that need to be excluded before setting the diagnosis of B-PSL. In contrast with pseudolymphoma, both PCMZL and PCFCL are more frequent in females (the female-to-male ratio being 2:1 and 1.5:1, respectively) and appear at later ages, concerning the mid-fifties and early-fifties, respectively. PCMZL shows a different body distribution, affecting mainly the upper trunk, followed by the upper extremities, and lastly the face, but PCFCL follows a similar pattern to that of PSL, with lesions being found mainly in the head and face, followed by the upper trunk. Additionally, PCMZL and PCFCL usually present as solitary or multiple dome-shaped papules, nodules, or erythematous plaques. The workup includes a thorough medical history, especially concerning exposure to one of the causes of B-PSL, along with a detailed clinical examination, including a thorough inspection of the patient and palpation of the lymph nodes, and laboratory blood count and serology tests, whose results are indicative of certain PSL causative agents [3]. A deep incisional biopsy or an excisional skin biopsy remains the hallmark of the diagnostic approach, as it is apparently based on the unique histopathological characteristics of the extracted tissue [2]. More specifically, cutaneous B-PSL nodular infiltrates affect mainly the reticular dermis and sometimes expand to the superficial subcutis. Said lesions consist of reactive germinal centers containing tingible body macrophages and small lymphocytes, which do not show any signs of atypia. Plasma cells, eosinophils, CD123+ dendritic cells, and a variety of T cells can also be found inside the lesion. PSL’s immunohistochemical properties CD19, CD20, CD79a, PAX-5, and bcl-6 positivity characterize the majority of the infiltrate’s B-cells, whereas CD21 positivity is indicative of the dendritic cells. Proliferation is significant mainly in the germinal centers based on Ki-67 and MIB-1 stains; there is polytypic Ig light chain production from the lesion’s plasma cells, and polyclonal Ig heavy chains are also identified [3]. Regarding differential diagnosis based on histology and immunohistochemistry, PCMZL and PCFCL, despite also affecting the reticular dermis and superficial subcutis and presenting some similar biomarkers, have certain distinct features. In PCMZL, plasma cells are more prominent and organized in the periphery of the infiltrate, and, most importantly, monotypic Ig light chains and monoclonal Ig heavy chains are found. In PCFCL, the presence of neoplastic follicles with centrocyte-like cells, scattered CD21+ dendritic cells, a lack of tingible body macrophages and proliferative activity, and B-cell clonality help in the distinction from PSL [3]. Still, histopathology alone does not provide a solid diagnosis of cutaneous pseudolymphoma [2,3,5,7].

Treatment options vary depending on the etiology and include the removal of the underlying causative agent and the prevention of re-exposure. Common drugs used in treating CBPL are topical or intralesional corticosteroids. Additional therapies include surgical excision, radiotherapy, and even cryotherapy [1,5,7]. Rarely, years after the diagnosis, patients have been described with a malignant transformation of CPSL, which makes long-term follow-up a necessity [5-7].

Herein, we present a case of a child with idiopathic CBPL that was surgically removed and sent over for biopsy in order for the diagnosis to be established.

## Case presentation

A 14-year-old male patient presented to the dermatologist with a two-month-old mass in the right lateral thoracic region. He stated that there is no pain or any other associated symptoms like itchiness, bleeding, or rash, and no other past medical history (including epilepsy) or family history that is of note. The child was also asked about allergies, piercings, or jewelry in that area, any recent or associated trauma, or any permanent or short-lasting tattoos, and he refused to have taken any recent medications or drugs. Despite that, he mentioned an insect bite near the area of interest in August, but he did not identify its species. As an adjunct, he has completed all the necessary vaccinations for his age frame, including the third dose of the COVID vaccine, which was done in September.

**Figure 1:**
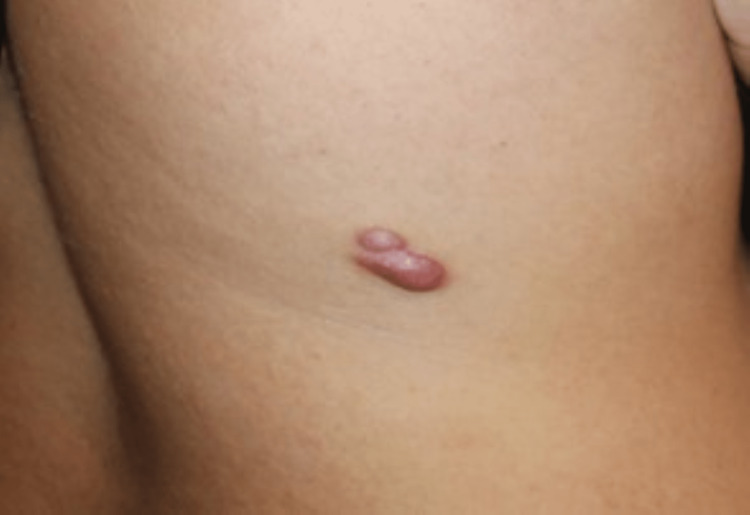
A firm, non-pigmented thoracic mass with fixed borders resembling to a mole in a 14-year-old male patient

During the examination, we did not detect any signs of folliculitis, and we observed a firm, non-pigmented mass with fixed borders, which, according to the patient, first appeared as a dark, flat spot resembling a mole (Figure 1).

**Figure 2:**
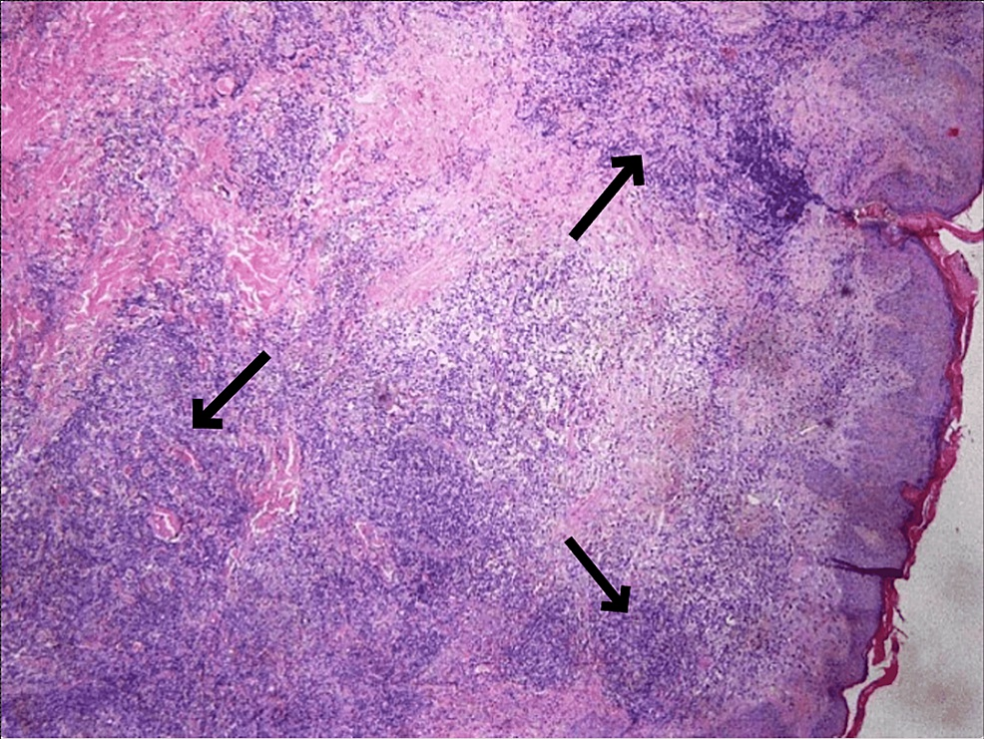
Dense lymphocytic infiltrates in the dermis consisting of small lymphocytes, presenting diffuse and nodular development pattern

**Figure 3:**
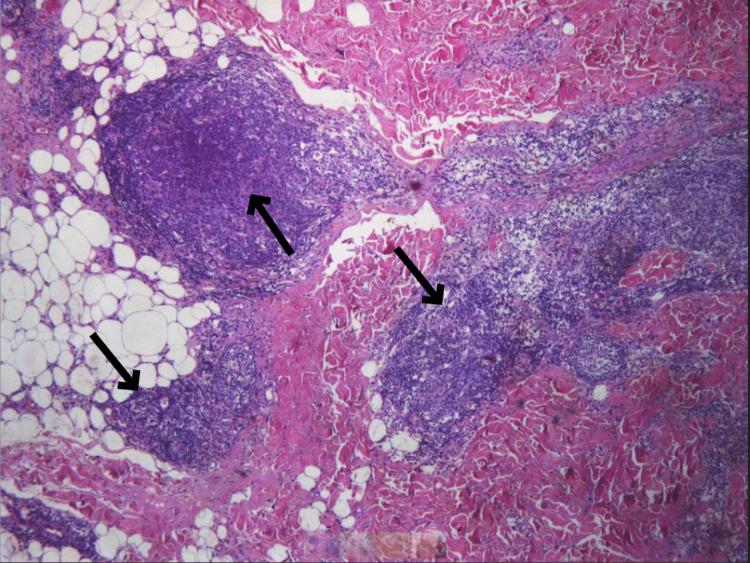
Dense lymphocytic infiltrates in the subcutis consisting of small lymphocytes, presenting diffuse and nodular development pattern

**Figure 4:**
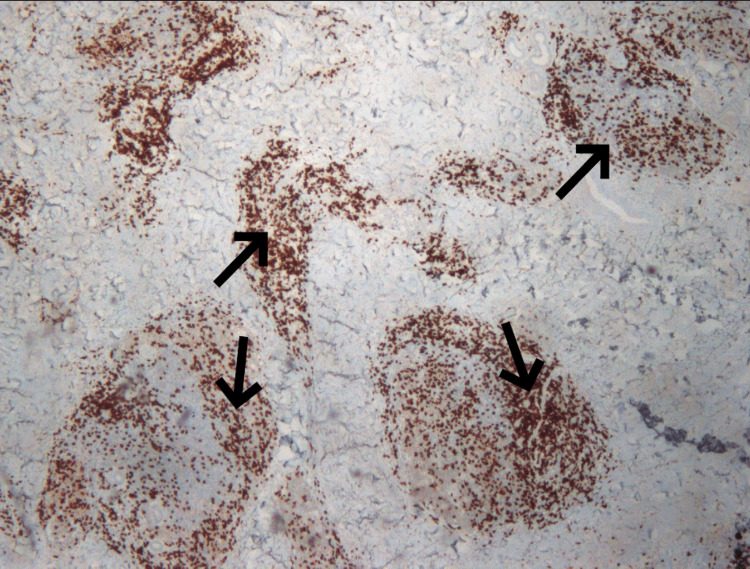
Pax5 stain showing B-lymphocytes allocated primarily in nodules, which correspond to reactive lymph follicles

A biopsy was taken for further investigation with the immunohistochemical markers CD20, CD79a, CIg, bcl-2, bcl-6, CD10, CD21, CD23, CD3, and Ki-67. The products of the biopsy were two paraffin cubes, two histological slides (H&E), and one histological slide Pax5, which, by the microscopic way of evaluation, displayed local hyperkeratosis and the presence of dense lymphocytic infiltration and more specifically spared the reticular dermis without the involvement of the epidermis (Figures 2-4).

**Figure 5:**
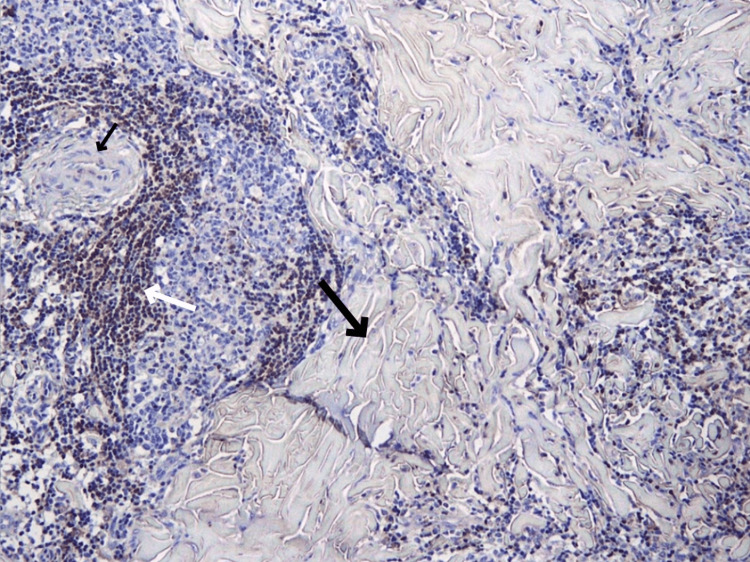
BCL-2 negative germinal centers (as shown by the small and big black arrows) in lymph follicles (positivity noticed only in mantle cells, as shown with the white arrow)

**Figure 6:**
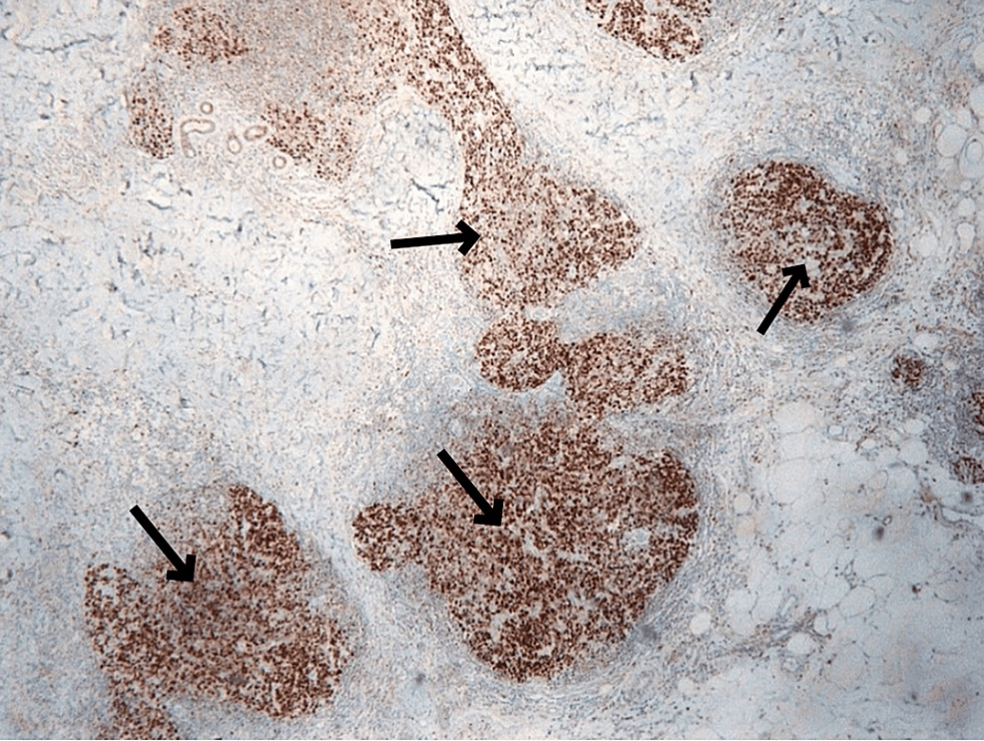
Homogenous expression of the marker BCL-6 stain in B-lymphocytes in the germinal centers

The immunophenotype of the specimens was B- and T-lymphocytes, with the former being bcl-2 negative (Figure 5) and bcl-6 and CD10 positive (Figure 6).

There was also the presence of polyclonal plasmacytes and a plethora of polymorphonuclear leukocytes with highly eosinophilic cytoplasm as the outer surface congregation. Taking the above into account, the diagnosis of cutaneous B-cell pseudolymphoma (cutaneous lymphoid hyperplasia) is confirmed due to a previous or persistent reaction to an immunological trigger.

**Figure 7:**
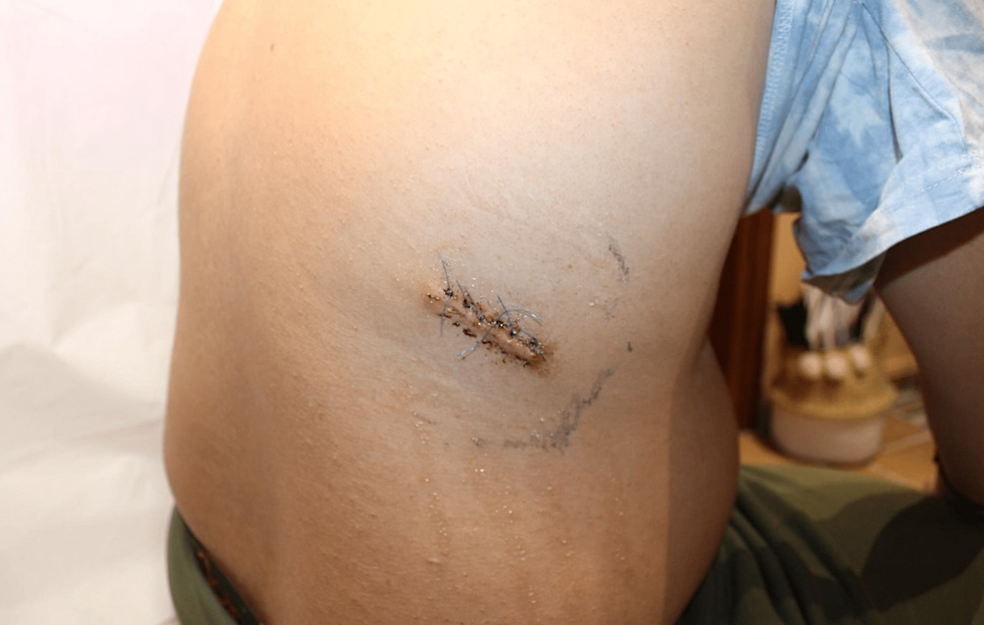
Site of the lesion in the lateral thoracic region (T7 dermatome level) after surgical excision has been performed

In such cases, the treatment considered is as follows: follow-up examinations, including frequent re-evaluation of the patient, due to the possibility of deterioration since B-pseudolymphoma has an erratic biological character that cannot exclude further antigenic reactions, which in turn could exacerbate the lesion, leading to either recurrence of a previous lesion or enlargement and worsening of an already existing one. All of this is done after surgical excision has taken place (Figure 7).

## Discussion

Cutaneous B-pseudolymphoma is an unusual skin disorder that is characterized by the presence of multiple or solitary small, red-brown papules and nodules on the skin. It is a benign, self-limited disorder, which means that if it is diagnosed early and treated, it does not usually cause any serious medical complications; in some rare cases, it has been documented that it resolves on its own, without treatment [4,6]. The exact cause of CPBL is unknown, but it is believed to be idiopathic or related to an immune system reaction due to a new stimulus. Our case was not able to be identified as a reaction to the insect bite on the ground because, according to the patient, the bite was not in the exact same area as the CBPL (it was a few centimeters away), and by the time the CBPL appeared, the skin irritation from the insect residue had resolved with a time difference of one month. However, it can be considered idiopathic due to the absence of other known precipitant stimuli. Such stimuli include a wide variety of drugs and foreign agents, including tattoo dyes, vaccinations, gold piercings, insect bites (which in our case was a suspected causative agent), and infections, mainly those caused by Borrelia burgdorferi and certain viruses [4]. The lack of these further supports our hypothesis of the idiopathic character of the present case. The case of interest is intriguing because of the worthily presented features that are not so common for the diagnosis of CBPL. The first feature is the site of the origin of the mass, in contrast with what has previously been described in the literature. More specifically, the lesion was located in the lateral thoracic region (T7 dermatome), whereas they are most commonly manifested in the face or, more rarely, medially of the thoracic region. Also, the age of the patient, 14 years old, is another interest-provoking characteristic of the case, as less than 10% of CBPL cases appear in children and adolescents [3]. This kind of lesion may be itchy, but it does not cause any other symptoms (the only exception being Borrelia-associated CBPL, which may present with local lymphadenopathy and Lyme disease symptoms), while in our patient there were no symptoms at all. Adding to that, the immunohistochemical properties of the case show interest. Positive results for the CD10 marker differ from the predominant CD19, CD20, and CD79a positivity that characterizes CBPL [3].

The current literature on this topic is rather limited, but both idiopathic and secondary presentations of the disease have been documented. Nnebe et al. record a case of seemingly idiopathic pseudolymphoma, appearing as a red mass on the scalp, as no exposure to possible causative agents was mentioned or realized, which showcased no other symptoms other than the mass and no abnormalities on test results. Special characteristics of the said case were also the age of the patient, being 12 years old, and the T-cell predominance (>90%) of the lesion described by matching biomarkers (CD3), which differentiates it from the case we are presenting. Surgical excision was performed after laboratory examinations, which also included a CT of the skull and a PET-CT scan, were done to ascertain the diagnosis, and no relapse was observed [8]. On the other hand, two cases of secondary PSL associated with Leishmania infection showcase infection-associated PSLs. Flaig and Rupec described a case of Leishmania donovani-associated PSL in the ear lobe in a 66-year-old female (age and sex are rarely associated with PSL). The lesion appeared suddenly as an erythematous patch, progressively increasing in size and causing swelling of the earlobe. Interestingly, the infiltrate was of a dual character, described as a mixed T- and B-cell PSL with CD20 and CD79a positivity but without clonality, leading to the diagnosis. Association with the amastigote was confirmed through the identification of DNA strains [9]. Vezzoli et al. report a Leishmania panamensis-associated PSL, the first of its kind, on a 66-year-old female. The disease first appeared on the upper right and lower left extremities and the forehead in the form of brown nodules. Histopathological examination of the lesion showed results matching CBPL, whereas sequencing of DNA fragments from the cells showed a match with Leishmania panamensis's genetic material. Treatment was successfully done using fluconazole and pentamidine [10]. Regarding drug-induced PSLs, Tian et al. report six independent cases of pseudolymphoma. Lack of other causes led to suspecting and also accusing the pharmaceutical treatment each patient was receiving before the onset of the disease as the inducing agent. Among the six cases, the presentation of the disease showed a minor degree of differentiation regarding location, duration, and histology, but all were successfully treated using either systemic or local glucocorticoids [11]. Lastly, although rare, there have been reports of cutaneous CBPL evolving into B-cell lymphoma. In one study of four cases, the transformation of one case of CBPL into a large B-cell lymphoma was documented. The exact mechanism of transformation from pseudolymphoma to lymphoma is not known, but it is believed that alterations in the tumor microenvironment, such as changes in the balance of cytokines and other immunomodulatory molecules, may play a role in this process. In addition, genetic and epigenetic alterations, such as mutations of oncogenes or silencing of tumor suppressor genes, may also be involved. For the aforementioned reason, the diagnosis was confirmed through a biopsy in the patient we presented, and due to the compact display of the mass, the total removal of it was decided as treatment. In some cases, however, topical corticosteroids or immunosuppressive drugs may be used first in order to reduce inflammation or prevent the recurrence of the lesions, but in idiopathic cases, CBPL tends to be more recalcitrant to topical and non-invasive treatments. Thus, surgical removal of the mass was the only definite option.

All in all, pseudolymphoma, although quite rare, is a present clinical entity affecting mainly the skin and one that closely imitates other important diseases, notably lymphoma. This case highlights PSL’s variability regarding prevalence and laboratory results, making it even more difficult to distinguish from other prominent pathologies. Thus, when suspected, the presence or absence of the disease should be aptly justified because it greatly impacts both the treatment and the patient's well-being.

## Conclusions

To conclude, CBPL is mostly due to an idiopathic cause. It usually appears on the face in the form of nodules, papules, and tumors. However, there are cases where CBPL appears in other parts of the body. In our case presentation, the patient examined was a 14-year-old boy who had a two-month-old mass in the right lateral thoracic region. Since there were no symptoms such as itchiness or rash, a biopsy was taken along with the surgical removal of the mass. Follow-up examinations were suggested so that further antigenic reactions would be prevented.
